# TRAF2 Knockdown in Nasopharyngeal Carcinoma Induced Cell Cycle Arrest and Enhanced the Sensitivity to Radiotherapy

**DOI:** 10.1155/2020/1641340

**Published:** 2020-05-30

**Authors:** Hongyuan Zhu, Weijun Ding, Jiaojiao Wu, Rongyou Ma, Zhaohu Pan, Xinli Mao

**Affiliations:** ^1^Department of Otolaryngology, Affiliated Tai Zhou Hospital of Wenzhou Medical University, No. 150 Ximen Road, Linhai, Taizhou 317000, China; ^2^Department of Gastroenterology, Affiliated Tai Zhou Hospital of Wenzhou Medical University, No. 150 Ximen Road, Linhai, Taizhou 317000, China

## Abstract

TRAF2 is a crucial adaptor protein involved in various signaling pathways. However, its biological functions in nasopharyngeal carcinoma (NPC) remain largely unknown. In the present study, we found that TRAF2 was overexpressed in nasopharyngeal carcinoma (NPC) cells. Knockdown of TRAF2 with shRNA significantly suppressed NPC cell proliferation and colony formation. The growth of xenograft tumor significantly reduced after TRAF2 was silenced. Conversely, the ectopic overexpression of TRAF2 significantly promoted cell proliferation and anchorage-independent growth. In TRAF2 knockdown cells, EGF-induced activation of transcriptional factors, including MSK1, CREB, and ATF2, markedly decreased. Accordingly, the transcriptional activity of AP-1 was substantially decreased in TRAF2-deficient cells. With the suppression of gene transcription, the expression of cyclin D1 was significantly impaired, which gave rise to the G0/G1 cell cycle arrest. Moreover, the overexpression of TRAF2 in NPC cells was associated with resistance to irradiation, and the potency of irradiation was substantially enhanced after TRAF2 was knocked down. Briefly, our studies demonstrated that TRAF2 had a crucial role in NPC development, and it might be of great potential to targeting TRAF2 for NPC prevention and treatment.

## 1. Introduction

TRAF2 belongs to the tumor necrosis factor receptor- (TNFR-) associated factor (TRAF) family, which is featured by the TRAF domain in its structure. So far, six representative members named TRAF1-6 and an atypical member TRAF7 have been characterized in mammalian cells. Generally, the typical TRAFs consist of a C-terminal TRAF domain and multiple zinc finger domains in the N-terminal. The TRAF domains have scaffolding activity and are engaged in the specificity of protein-protein interaction, such as the oligomerization of TRAFs and the interactions between upstream mediators and downstream effectors [1]. Moreover, except TRAF1, other members of the TRAF family contain the RING finger domain in the N-terminal, which is well known for its function in protein ubiquitination. Some studies have shown that TRAF2 possessed lysine (K) 63-specific E3 functions [2], but the biological function of its E3 activity is still elusive.

As an adaptor protein, the role of TRAF2 in TNF-induced signaling is well documented. Upon TNF binding, TRAF2 is recruited to activated TNFR1 and TNFR2 and engaged in signal transduction, resulting in the activation of downstream signaling, including the canonical NF-*κ*B pathway and JNK/p38 pathway [3, 4]. Despite the well-known role in TNF-TNFR signaling, recent studies demonstrated that TRAF2 is involved in other receptor-mediated pathways. For instance, TRAF2 is required for the activation of NF-*κ*B induced by nucleotide-binding oligomerization domain- (NOD-) like receptors [5]. In RIG-I like receptor- (RLR-) mediated antiviral responses, depletion of TRAF2 results in reduced expression of interferon (IFN) and proinflammatory cytokines [6]. Even in cytokine receptor signaling, including IL-17 receptors and IFN receptor, TRAF2 also plays critical roles in the regulation of the immune system and apoptosis [7]. Increasing evidence suggested the overexpression of TRAF2 was closely correlated with cancer development. In gastric cancer, it was found that high TRAF2 expression was associated with tumor invasion and metastasis and was an important prognostic indicator [8]. Beyond that, in other different tumors, such as prostate cancer, breast cancer, and diffuse large B-cell lymphomas (DLBCL), altered TRAF2 expression was significantly related to poor survival time [9–11].

According to the statistics, in China, there were 42,100 new cases and 21,320 deaths from nasopharyngeal cancer in 2013. Most of the nasopharyngeal carcinoma is low or undifferentiated squamous carcinoma, which is characterized by high malignancy, rapid growth, and distant metastasis [12]. Epidemiology studies revealed that the occurrence of NPC is closely related to EBV infection [13]. Some studies reported that TRAF2 was involved in EBV-associated nasopharyngeal carcinoma development. Through interacting with the viral oncoprotein LMP1, TRAF2 induced K63-linked ubiquitination of p53 and promoted p53 stabilization. Moreover, TRAF2 also had a critical role in the Epstein-Barr virus BRFF1 protein, a Na-induced lytic infection by activating Jun N-terminal protein kinase (JNK) [14]. In the present study, we examined the expression of TRAF2 in NPC cells and adopted shRNA to silence TRAF2 in NPC cells and then studied the effects of TRAF2 knockdown on NPC cells. Furthermore, we investigated the signaling pathway mediated by TRAF2 in NPC cells.

## 2. Material and Methods

### 2.1. Cell Lines and Reagents

Human immortalized nasopharyngeal epithelial cell NP460 and nasopharyngeal carcinoma cell lines, including CNE1, CNE2, C666-1, HNE1, HNE3, HK1, HNE2, HONE1, and SUNE1, were obtained from the Cell Bank of Chinese Academy of Sciences (Shanghai). The 293T cells for virus infection was purchased from American Type Culture Collection (ATCC, Manassas, VA). All cells were cultured by following the standard protocols. The primary antibodies including anti-TRAF2, p-MSK1, p-CREB, cyclin D1, CDK4, cyclin A, CDK2, cyclin B, CDK1, p27, p21, *β*-actin, Ki67, cleaved PARP, cleaved caspase-3, and secondary HRP-conjugated goat anti-mouse/rabbit IgG antibodies were products of Cell Signaling Technology (Danvers, MA). The SuperSignal™ West Dura Extended Duration Substrate was a product of Thermo Fisher Scientific (Waltham, MA).

### 2.2. MTS Assay

NPC cells stably transfected with TRAF2-shRNA (3 × 10^3^/well) were plated into a 96-well plate and maintained in the incubator for various time points. To examine cell proliferation, 100 *μ*l/well CellTiter96 Aqueous One Solution (Promega, Madison, WI) was added and incubated at room temperature for 15 mins. The absorbance was measured by following the standard procedures.

### 2.3. Colony Formation Assay

After digestion with trypsin, tumor cells (8 × 10^3^) were harvested and suspended with 1.5 ml BME (basal medium eagle)/10% FBS/0.33% agar mixture. Then the cell suspension was plated into a 6-well plate coated with solidified BME (basal medium eagle)/10% FBS/0.5% agar. The plate was placed in a 5% CO_2_ incubator at 37°C for 1-2 weeks. The number of cell colonies formed in the agar was counted using the microscope.

### 2.4. Flow Cytometry Analysis

The cells were seeded in a 6-well plate and cultured overnight; then the culture medium was replaced with the basic medium containing 0.1% FBS for about 24 h to induce G0/G1 cell cycle arrest. After that, the cells were incubated with completed culture medium containing 10% FBS for another 24 h. After digestion, the cells were collected and fixed with cooled 70% ethanol. Washing with ice-cold PBS, the fixed cells were stained with PI. After the incubation with DNase-free RNase A at room temperature for 15 mins, the stained cells were subjected to cell cycle analysis by flow cytometry. For Annexin V-PI double staining, after transfection, the cells were exposed to 2 Gy irradiation for 24 hours and then digested with trypsin; cell suspension was collected and washed with PBS for about three times, then incubated with 5 *μ*l Annexin V and Propidium Iodide (Cat. V13242, Thermo Fisher) at room temperature for 15 mins, and then analyzed by flow cytometry.

### 2.5. Lentivirus Infection

To generate TRAF2 stable cells, PSPAX2 and PMD2-G plasmids (Addgene) were cotransfected into 293T cells with pLKO.1-shTRAF2 plasmids (TRCN0000004571, TRCN0000367433, GE horizon). The cell culture medium containing a viral supernatant was collected after 48 hours and filtered with a 0.45 mm filter. For virus infection, the NPC cells were incubated with the viral supernatant together with 10 mg/ml polybrene overnight. The infected cells were maintained in a fresh medium for another 24 h. Puromycin was used for stable cell selection.

### 2.6. Immunoblot Analysis

The cells were lysed using the RIPA lysis buffer (#89900, Thermo Fisher Scientific) supplied with proteinase inhibitors, and protein concentrations were determined by the BCA protein assay (Pierce, Rockford, IL, USA). In all, 30 *μ*g of cellular protein was subjected to SDS-PAGE, followed by transferring to PVDF membranes. Blocking with 0.5% nonfat milk solution, the membranes were incubated with indicated primary and corresponding second antibody in sequence, and the probed protein on the membrane was visualized using the ECL substrate (#32132, Thermo Fisher Scientific).

### 2.7. Real-Time PCR

The tumor cells were starved overnight and then stimulated with 30 ng/ml EGF for 8 h; the cells were harvested and total RNA was extracted with TRIzol™ Reagent (Cat. #15596018, Thermo Fisher) by following the standard protocols. Five micrograms of RNA was used to synthesize cDNA with Superscript TMII Reverse Transcriptase (Cat. #18064014, Thermo Fisher). The qPCR reactions, including SYBR Premix Ex Taq II (Cat. #RR82LR, Takada), were performed in the Applied Biosystems 7500 Real-Time PCR systems. The 18S was loaded as internal control. The following primers were used in the studies. 18S (F): 5′-CCCGGGGAGGTAGTGACGAAAAAT-3′; 18S (R): 5′-CGCCCGCCCGCTCCCAAGAT-3′. CCND1 (F): 5′-CCGCTCGAGTCCAGGAGCAGATCGAA-3′; CCND1 (R): 5′-AAGGAAAAGCGGCCGCAGCCCAGGAAGCAAAAGA-3′.

### 2.8. AP-1 Transcriptional Activity Assay

The transcriptional activity was measured with the AP-1 Reporter Kit (Cat. #60612, Bioscience) by following the instructions. Briefly, shGFP and shTRAF2 stable cells (1 × 10^5^) were seeded into a 24-well plate and cultured about 90% confluent before transfection. The cells were transfected with AP-1 luciferase reporter vector by Lipofectamine 2000 and cultured for 48 h. Then the cell culture medium was changed to the assay medium containing 30 ng/ml EGF (PHG0315, Thermo Fisher) and incubated at 37°C for about 8 h. The activities of luciferase in tumor cells were determined with the Dual Luciferase (Firefly-Renilla) Assay System (Cat. #60683, Bioscience) according to the protocols.

### 2.9. Xenograft Mouse Model

The in vivo animal experiment was performed under the approval of the Animal Ethics Committee of Wenzhou Medical University. CNE1-shGFP and CNE1-shTRAF2 (2 × 10^6^/100 *μ*l) stable cells were injected subcutaneously into the right flank of 6-week-old nude mice (*n* = 5). The tumor volume was monitored every three days using a caliper. Tumor volume was calculated as length × (width)^2^/2. When the tumor volume reached 1000 mm^3^, the mice were sacrificed and the xenografts were photographed and weighed.

### 2.10. Immunohistochemistry Staining

Briefly, xenograft tumors were fixed with formalin and embedded with paraffin. The sliced tissues were deparaffinized, rehydrated, and unmasked by immersing into the boiling sodium citrate buffer (10 mM, pH 6.0) for 10 mins, followed by raising with PBS two times. The slides were treated with 3% H_2_O_2_ in methanol for 10 mins and washed with PBS twice; 50% goat serum solution was used for unspecific antigen blockade, followed by incubation with primary antibodies in a humidified chamber at 4°C overnight. After being hybridized with the secondary antibody, the slides were incubated with the VECTASTAIN Elite ABC kit and visualized using the HRP substrates. After staining with hematoxylin, the slides were dehydrated and sealed. Eight random fields on the slides were selected and the intensity of indicated markers were analyzed using the Image-Pro Plus (v.6) software.

### 2.11. Statistical Analysis

The SPSS software (version 16.0) was employed for statistical analysis. Each experiment was performed at least 3 times and the quantitative data was calculated as mean ± SD. One-way ANOVA was adopted to analyze statistical differences and *p* < 0.05 was considered to be a significant difference.

## 3. Results

### 3.1. TRAF2 Played a Crucial Role in Nasopharyngeal Carcinoma (NPC) Cells

**Figure 1:**
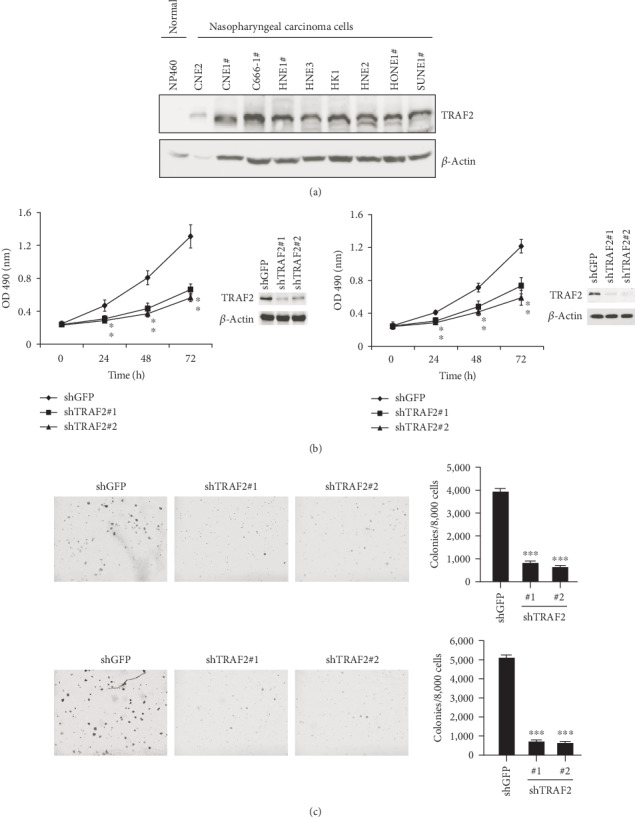
TRAF2 played a crucial role in NPC cells. (a) The expression of TRAF2 in normal nasopharyngeal epithelial cell NP460 and nine NPC cells was examined by western blotting. (b) TRAF2 knockdown inhibited NPC cell proliferation. TRAF2 deficient CNE1 (left) or HK-1(right) cells were established with shRNA as described, and the cell proliferation rate at different time points was examined by MTS assays. The efficiency of shRNA was confirmed by western blotting. (c) TRAF2-shRNA suppressed the NPC colony formation in soft agar. CNE1 (upper) or HK-1 (below) cells transfected with shRNA and the anchorage-independent growth assays were performed as described. Left: the representative images, right: quantitative data presented as mean ± SD.^∗^*p* < 0.05, ^∗∗∗^*p* < 0.001 vs. control. ^#^The cells that harbored EBV viral sequences.

Firstly, we examined the expression of TRAF2 in NPC cells by western blotting. As the results are shown in Figure 1(a), in contrast with the normal nasopharyngeal epithelial cell NP460, TRAF2 expression was dramatically elevated in all tested NPC cells. To verify the role of TRAF2, we developed shRNA to knockdown TRAF2 expression. As demonstrated in Figure 1(b), the shRNA we used significantly knockdown the expression of TRAF2 in NPC cells. With the silence of TRAF2, the proliferative abilities of NPC cells were substantially decreased. Comparing with the control group, the proliferation rate of TRAF2 deficient cells decreased about 50%. Anchorage-independent growth is an important characteristic of malignant tumor cells, so we employed soft agar assays to check the effects of shRNA on colony formation. As shown in Figure 1(c), after TRAF2 was knocked down, the abilities of NPC cells to form clones were dramatically inhibited, and the number of clones formed in soft agar was markedly decreased, with a reduction of 73% and 82% in CNE1 and HK-1 cells, respectively.

### 3.2. TRAF2-shRNA Inhibited Tumor Growth in Nude Mice

**Figure 2:**
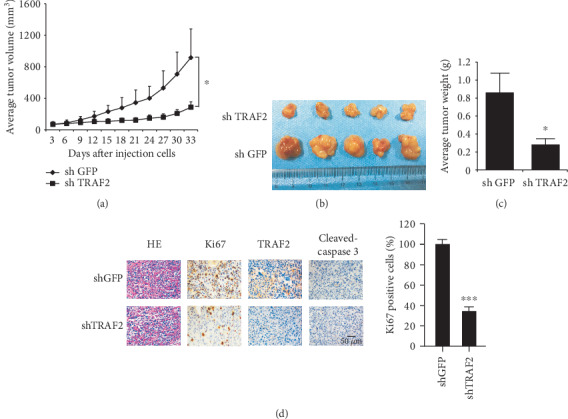
TRAF2 shRNA impaired tumor growth in nude mice. (a–c) CNE-1 cells transfected with shGFP or shTRAF2 were injected into nude mice, and the growth curve of xenografts (a), the photograph of xenografts (b), and the weight of xenografts (c) were examined. (d) Ki67 and TRAF2 expression in tumor tissue. The expressions of Ki67, TRAF2, and cleaved caspase-3 in xenografts were detected by immunohistochemistry as described. Left: the representative images. Right: the expression of Ki67; cleaved caspase-3 was quantified. ^∗∗∗^*p* < 0.001 vs. vehicle.

To further confirm the role of TRAF2 in NPC cells, we injected TRAF2-deficient CNE1 cells into nude mice and examined the tumorigenicity ability of TRAF2-shRNA cells. As shown in Figures 2(a)–2(c), after TRAF2 was silenced, the tumor growth of CNE1 cells was significantly suppressed. 33 days after inoculation, the average tumor volume in the shGFP group has reached about 1000 mm^3^, whereas the volume in the TRAF2 knockdown group was only around 300 mm^3^. Meanwhile, the tumor weight was also substantially different (0.83 g vs 0.28 g). Immunohistochemistry staining demonstrated that in TRAF2-shRNA tumor tissue, the expression of Ki67, which is a well-known biomarker to predict cell proliferative potentials, significantly decreased, implying NPC cell proliferative capabilities were impaired after TRAF2 was silenced. Moreover, we also checked the expression of cleaved caspase-3, which is indicative of cell apoptosis, and no significant difference was observed (Figure 2(d)).

### 3.3. TRAF2 Overexpression Promoted Nasopharyngeal Epithelial Cell Transformation

**Figure 3:**
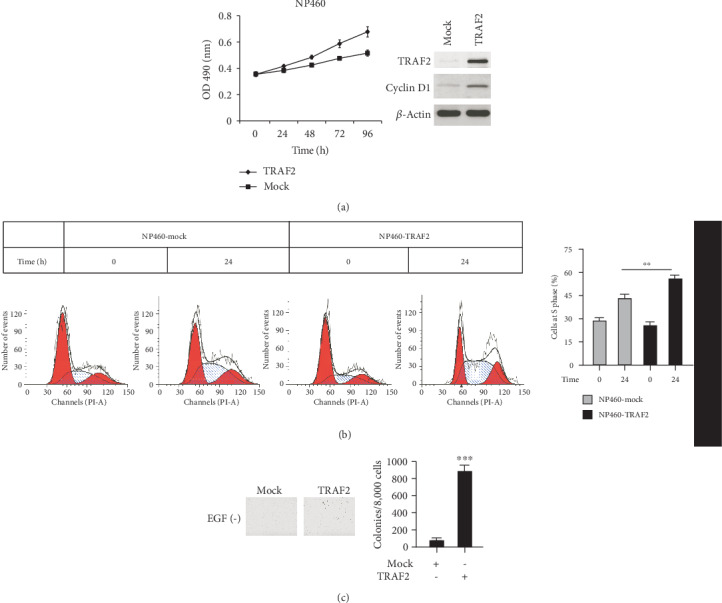
TRAF2 overexpression promoted normal nasopharyngeal cell transformation. NP460 cells were transfected with pcDNA3.1 plasmids containing TRAF2, and stable cell lines were selected with G418. (a) The proliferative ability of TRAF2-transfected cells was examined. (b) The cells were starved overnight and then cultured with medium supplemented with 10% serum; the cell phase distribution was analyzed by flow cytometry. (c) The colony formation abilities of TRAF2-transfected cells were examined as described. ^∗∗^*p* < 0.01, ^∗∗∗^*p* < 0.001 vs. control.

On the other hand, in NP460 cells, ectopic overexpression of TRAF2 was performed by plasmid transfection and the effects were examined. In terms of the effects on cell proliferation, TRAF2 overexpression in NP460 cell substantially improved the proliferation rate, the growth rate increased about 30%, and the expression of cyclin D1 significantly increased in TRAF2-overexpressing cells. In addition, the results of flow cytometry analysis showed in contrast with the mock group, the proportion of cells in S phase significantly increased in the TRAF2-overexpressing group (Figure 3(b)). Beyond that, we found that TRAF2 overexpression promotes the transformation of normal NP460 cells. Generally, normal cells do not have the ability to form clones in soft agar. However, after TRAF2 was overexpressed in NP460 cells, without the stimulation of EGF, a large number of cell clones were observed (Figure 3(c)). The results suggested that TRAF2 may have a crucial role in NPC development.

### 3.4. TRAF2-shRNA Resulted in G0/G1 Cell Cycle Arrest

**Figure 4:**
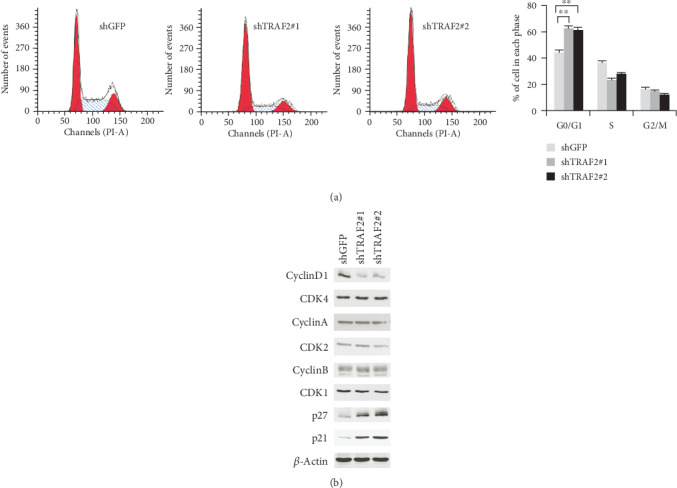
TRAF2 silence resulted in G0/G1 cell cycle arrest. (a) After shRNA transfection, the cells were fixed and stained with PI, and the cell phase distribution was analyzed by flow cytometry. (b) The expression of cyclins and cyclin-dependent kinase in TRAF2 knockdown cells were examined by western blotting. ^∗∗^*p* < 0.01 vs. control.

Due to the effects on cell proliferation, we have analyzed the effect of TRAF2-shRNA on cell cycle distribution. As shown in Figure 4(a), in the control group, the proportion of cells in G0/G1 phase was about 40%, and in TRAF2-deficient cells, the percentage of G0/G1 cells went up to 60%, proving that the decrease of TRAF2 in NPC cells resulted in G0/G1 arrest. Next, the changes of cyclins and cyclin-dependent kinases (CDKs) engaged in cell cycle regulation were examined in TRAF2 knockdown cells. The results showed that the expression of cyclin D1, which promotes G0/G1 progression, significantly decreased (Figure 4(b)). In contrast, other cyclins, such as cyclin A and cyclin B, which are involved in G1/S and G2/M phase transition, respectively, had no obvious changes. In addition, in TRAF2 knockdown cells, the expression of p21 and p27, which also play an important role in G0/G1 cell cycle regulation, substantially elevated.

### 3.5. TRAF2 Was Involved in the Mediation of AP-1 Transcriptional Activity

**Figure 5:**
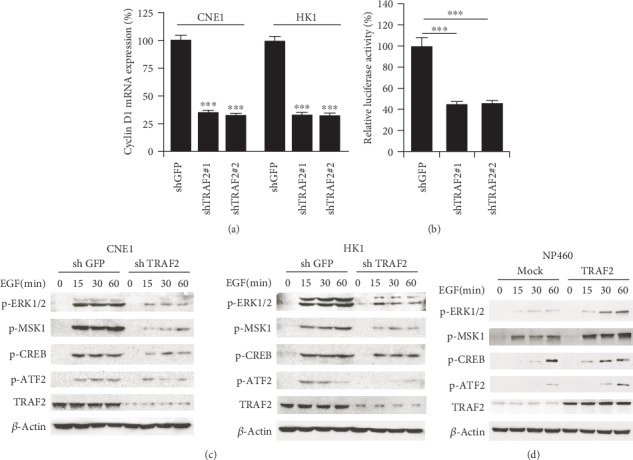
TRAF2 was involved in the mediation of AP-1 transcription activity. (a) Cyclin D1 mRNA was in TRAF2 knockdown CNE-1(left) and HK-1 (right) cells. (b) TRAF2 knockdown inhibited AP-1 transcriptional activity. AP-1 luciferase reporter was transfected into TRAF2-deficient CNE1 cells, and the activity of luciferase was determined as described. ^∗∗∗^*p* < 0.001 vs. control. (c, d) TRAF2 mediated the activities of transcription factors. TRAF2-deficient CNE1 or HK-1 cells (c) or TRAF2-overexpression NP460 cells (d) were starved overnight and then stimulated with EGF at different time points; cell lysates were collected subjected to western blotting, and the expression of the indicated protein was probed with the corresponding antibody.

To clarify the mechanisms by which cyclin D1 was downregulated in TRAF2-deficient NPC cells, qPCR was carried out to examine the mRNA levels of cyclin D1. As demonstrated in Figure 5(a), in TRAF2-shRNA-transfected cells, the mRNA of cyclin D1 was substantially decreased, suggesting that the decrease of cyclin D1 was attributed to the inhibition of gene transcription. AP-1 is the well-known transcriptional factor responsible for CCND1 gene transcription. To validate the effect of TRAF2 on AP-1 transcriptional activity, AP-1 luciferase reporter vector was transfected into the NPC cells, and the transcriptional activity of AP-1 was detected. The results showed that in contrast with the control, the activity of AP-1 in TRAF2 knockdown cells dramatically declined, with a decrease of about 57% (Figure 5(b)). Further investigations demonstrated in the TRAF2-shRNA NPC cells found that EGF-induced activation of ERK1/2 significantly decreased. With inhibition of ERK1/2 activation, the activities of its downstream transcriptional factors, such as MSK1, CREB, and ATF2, which are engaged in the regulation of AP-1 activities, also significantly decreased (Figure 5(c)). On the contrary, in TRAF2-overexpressing NP460 cells, EGF-stimulated activation of ERK1/2 and its downstream transcriptional factors were substantially enhanced (Figure 5(d)). All these dates suggested the signal pathways mediated by TRAF2 were involved in the regulation of AP-1 transcription activity.

### 3.6. TRAF2 Knockdown Enhanced the NPC Sensitivity to Radiotherapy

**Figure 6:**
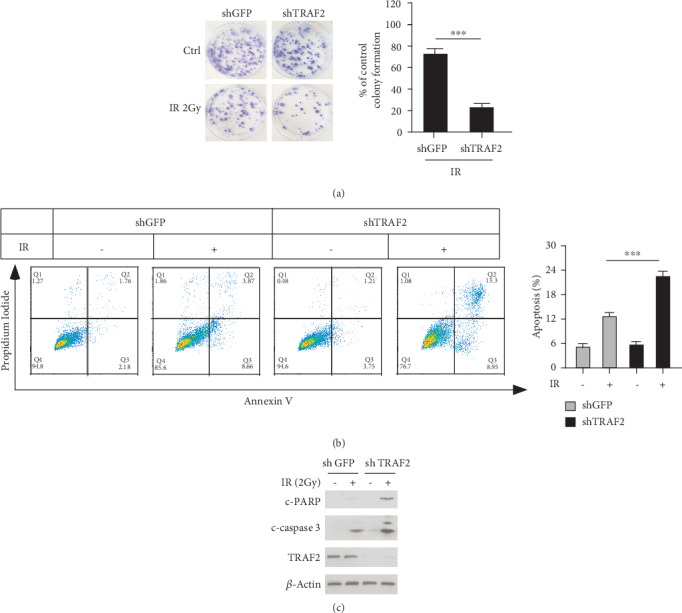
TRAF2 enhanced the sensitivity of NPC cells to radiation. (a) TRAF2 knockdown decreased the clonogenic survival of NPC cells after radiation. CNE1 cells were transfected with shGFP or shTRAF2 and then subjected to 2 Gy irradiation for 48 h, and then clonogenic survival assay was performed as described. Left: the representative images. Right: the quantitative data as mean ± SD. ^∗∗∗^*p* < 0.001 vs. control. (b, c) TRAF2 silencing enhanced irradiation-induced cell apoptosis. After shRNA transfection, CNE1 cells were treated with 2 Gy irradiation for 24 h, and then the cells were divided into two parts, one for Annexin V/Propidium Iodide double staining as described in Material and Methods (b); the lysates of the other part were collected, and the cleaved PARP and caspase-3 were detected by western blotting (c).

Radiation is a standard therapy for NPC treatment, and we also evaluated the effects of TRAF2 knockdown on the NPC sensitivity to radiotherapy. As shown in Figure 6(a), in the vehicle group, after 2 Gy irradiation (IR) for 48 h, the clonogenic survival of NPC cells was moderately decreased, while after NPC cells were transfected with TRAF2-shRNA, the survival of NPC cells dramatically declined (82% vs. 30%), suggesting the high expression of TRAF2 may be associated with the resistance to radiation. The results of Annexin V/Propidium Iodide double staining demonstrated that in TRAF2 deficient cells, after irradiation, the ratio of Annexin V positive cells was substantially increased (23% vs 12%), suggesting irradiation-induced cell apoptosis, significantly improved. Moreover, we also detected that the expression of two makers of cell apoptosis, cleaved PARP and cleaved caspase-3, slightly increased; in contrast with the control cells, irradiation-induced expression of cleaved PARP and caspase-3 in TRAF2 knockdown cells markedly increased (Figure 6(b) and 6(c)).

## 4. Discussion

As an essential adaptor protein involved in cell signaling transduction, a large number of literature has revealed that TRAF2 was involved in tumor development and progression [15, 16]. However, the expression of TRAF2 and its role in nasopharyngeal carcinoma are less known. Zeng et al. reported that in contrast with the nontumor nasopharyngeal epithelial tissues, the mRNA level of TRAF2 in nasopharyngeal carcinoma significantly elevated through gene expression profiling analysis [17]. Consistently, in our studies, the western blotting results showed that compared to the normal nasopharyngeal epithelial cell NP460, the TRAF2 protein levels significantly increased in all examined NPC cells (Figure 1(a)). So far, the underlying mechanisms by which TRAF2 was upregulated in NPC is not clear, and the EBV infection seems to be one of the reasons. Guasparri et al. demonstrated that in EBV-associated lymphoid malignancies, the latent membrane protein-1 (LMP1) of EBV cooperated with LMP2A to upregulate TRAF2 expression on transcriptional levels which leads to the activation of NF-*κ*B [18]. Indeed, in EBV-positive NPC cell lines such as C666-1 and HONE-1, TRAF2 overexpression was observed. However, the increase of TRAF2 in EBV negative cells suggested that there are other mechanisms involved in the regulation of TRAF2 expression, and further investigations are needed to elaborate on related mechanisms.

To determine the role of TRAF2 in NPC cells, we silenced TRAF2 expression by shRNA. After TRAF2 was knocked down, both the cell proliferation and colony formation of NPC cells are significantly suppressed, and the tumorigenesis and tumor growth rate in nude mice are substantially decreased. By contrast, when TRAF2 was overexpressed in normal nasopharyngeal epithelial cells, the proliferative potential and anchorage-independent growth abilities significantly increased. All these results demonstrated that TRAF2 had a crucial role in nasopharyngeal carcinoma development. In TRAF2 knockout NPC cells, the proportion of cells in the G0/G1 phase was significantly higher than control cells, suggesting that the signaling pathway mediated by TRAF2 was involved in cell cycle regulation. Cyclins are a class of proteins that play essential roles in cell cycle regulation, especially cyclin D1, by forming a complex with cyclin-dependent kinase 4/6 and inducing its activation, driving cells through the G0/G1 phase. In TRAF2 knockdown cells, the expression of cyclin D1 dramatically decreased, which was considered to be contributed to the G0/G1 arrest. In tumor cells, due to the difference in a cellular context and signaling pathway, the cyclin D1 expression is often mediated by different transcription factors. Multiple factors, including AP-1, Sp1, and CREB, signal transducers, and activators of transcription 3/5 (STAT3/5) and Kruppel-like factor-8 (KLF-8) were found to be engaged in cyclin D1 regulation [19–21]. In our studies, the results revealed that the phosphorylation of transcription factors, such as CREB and ATF-2, which were involved in the regulation of AP-1 activity, substantially decreased in TRAF2 knockdown cells. Conversely, in TRAF2-transformed NP460 cells, their activities were significantly boosted, implying that these factors are involved in the regulation of pathways mediated by TRAF2. Furthermore, the reporter luciferase assay further confirmed that the transcriptional activity of AP-1 dramatically decreased in TRAF2-deficient cells. Therefore, we thought that the inhibition of cell proliferation caused by TRAF2 knockdown was mainly due to the suppression of AP-1 transcriptional activity and subsequent decrease of cyclin D1. It is best studied that the mitogen-activated protein kinase (MAPK) pathway stimulates the activity of AP-1, in TRAF2-deficient NPC cells, and the decrease of ERK1/2 phosphorylation was observed. In addition, some studies have disclosed that the NF-*κ*B pathway was also involved in the regulation of AP-1 activity [22]. Given the critical role of TRAF2 in the NF-*κ*B signaling pathway, it cannot exclude the possibility that TRAF2 regulates AP-1 transcriptional activity by affecting the NF-*κ*B signaling pathway.

As a hub scaffold protein in signal transduction, especially the role in the regulation of NF-*κ*B activity, abnormal expression of TRAF2 is often related to tumor resistance. It has been demonstrated that in FLT3-ITD-positive AML cells, TRAF2 contributed to TNF-*α* resistance [23]. Independent of its role in NF-*κ*B signaling, TRAF2 was found to be able to interact with the proteins of the inhibitor-of-apoptosis protein (IAP) family and antagonize cell apoptosis [24, 25]. Gonzalvez et al. showed that TRAF2 directly mediated the K48-linked polyubiquitination of caspase-8, which led to its degradation and inactivation of cell apoptosis [26]. In the clinic, radiotherapy is the primary choice for early nasopharyngeal carcinoma. However, advanced nasopharyngeal carcinoma is less sensitive and is prone to resistance. The underlying mechanisms by which NPC cells exert resistance to radiation are mainly unknown. In TRAF2 knockdown NPC cells, radiation-induced cell apoptosis significantly increased, suggesting that TRAF2 overexpression may be related to it and there is a synergy between radiation and TRAF2-targeted therapy.

In summary, we demonstrated that TRAF2 was overexpressed in NPC cells and played a crucial role in NPC development. By mediating the signaling pathways involved in the regulation of AP-1 transcriptional activities, TRAF2 knockdown substantially impaired NPC cell proliferation and strengthened the sensitivities to radiotherapy. Our studies suggested that TRAF2 might be a potential molecular target for NPC prevention and treatment.

## Data Availability

The data used to support the findings of this study are included in the article.
